# The Role of the Clinical Exercise Physiologist in Reducing the Burden of Chronic Disease in New Zealand

**DOI:** 10.3390/ijerph18030859

**Published:** 2021-01-20

**Authors:** Amy Pearce, Glynis Longhurst

**Affiliations:** Wintec, Centre for Sport Science and Human Performance, Private Bag 3036, Hamilton 3240, New Zealand

**Keywords:** clinical exercise physiology, non-communicable disease, health economics, health and wellness

## Abstract

Clinical exercise physiologists (CEPs) specialize in managing long-term, non-communicable health conditions using scientific rehabilitative exercise prescription, which alleviates the burden of these conditions on health care systems. This is evident, particularly in Australia (AUS), where they are registered as health care workers. CEPs have been shown to reduce the physical burden of long-term conditions on populations and the economic load that these place on national health departments. This article aims to evidence the effectiveness of CEPs in Noncommunicable Disease (NCD) rehabilitation, the cost-effectiveness of supervised exercise prescription for various NCDs by CEPs in AUS, and related cost-effectiveness New Zealand (NZ) burden of disease. This article highlights the important role NZ. CEPs can play in reducing chronic disease cost if given the same opportunities as Australian CEPs within NZ’s health care system.

## 1. Introduction

A CEP holds a minimum of a 3 year Bachelors Degree in Science and a postgraduate qualification, either a one year Postgraduate Diploma or a 2 year Masters Degree specializing in clinical Exercise Physiology. They are allied health professionals specializing in the delivery of scientific exercise interventions for people with acute, subacute, or chronic medical conditions across a wide spectrum of health, including but not limited to cardiovascular, respiratory, endocrine, musculoskeletal, mental and neurological disease. In order to register as a CEP, the practitioner must meet the standards expected of practicing CEPs as per the Standards Met by Registered CEPs in NZ document of 2015, and complete 500 h of documented clinical experience under the supervision of a registered CEP within a tertiary education environment while registered for the postgraduate course. Once the hours and qualification have been completed the CEP must sit the Clinical Exercise Physiology Board of NZ competency exam. If successful, the CEP may then register with the Clinical Physiology Registration Board (CPRB) as a Registered Clinical Exercise Physiologist (RCEP) to maintain clinical competency and receive their annual practicing certificate. These registrations are similar internationally with the American College of Sports Medicine-CEP registration, Canadian Society for Exercise Physiology, South African Biokinetics Association and Exercise & Sport Science AUS-Accredited Exercise Physiologist, and the British Association of Sport and Exercise Science-CEP.

This article attempts to quantify the burden of NCD within NZ and how the vastly underutilized CEP is the most qualified member of the multidisciplinary team to manage and prevent these conditions using scientific exercise prescription.

Studies indicate that CEPs provide effective multidisciplinary care, and Gillam noted that exercise advice given to patients by physicians in primary care settings was translated more effectively by CEPs, which motivated people with chronic conditions to exercise [1]. A systematic review by Schuch et al., noted that the multidisciplinary treatment of people with severe mental illness should focus on improving cardiovascular fitness to improve all-cause mortality, and recommended that qualified professionals that could support these patients with exercise therapy, be included as part of the multidisciplinary team in mental health treatment [2]. According to Soan et al., cardiac programs designed and facilitated by CEPs were extremely successful in altering exercise patterns and physical behaviors. They also helped prevent or delay subsequent cardiac arrest, improved exercise tolerance, circulation, and muscle atrophy, significantly reduced risk factors for comorbidities, and improved quality of life [3]. Understanding the current global burden of NCDs indicates the massive cost that these chronic conditions are placing on the world. NZ is a small, high income country with its own enormous burden of NCDs. This article will discuss the implications of the financial cost of these conditions as well as evidence that exercise intervention strategies by CEPs have reduced the financial impact of cardiovascular disease (CVD) including ischaemic heart disease and stroke, mental illness (particularly depression) and type 2 diabetes mellitus (T2DM) in AUS, and that there is the potential for the same to occur in NZ if the government will recognize this profession as the key to cost-effective models of prevention and treatment of NCDs.

Australian CEPs have been providing exercise-related services funded by Medicare AUS since 2006. Medicare is AUS’s universal health care scheme, which provides Australian residents access to health care. The integration of CEP’s was an essential step in effecting a hands-on healthcare model focused on prevention. This includes timely detection and treatment of the significant risk factors of inactivity, obesity, and metabolic risk [4]. Medicare rebates allow for five sessions with a qualified CEP. CEPs have been proven to reduce overall health costs in certain long- term, chronic conditions. However, there is no government-assisted funding for CEPs in NZ where the burden of NCDs is extremely high. In South Africa, CEPs are healthcare providers registered with the Health Professions Council of South Africa, in the same allied health category as physiotherapists and podiatrists, since 1983.

## 2. Global Burden of NCDs

Between 2010 and 2030, the cost of Non-Communicable Disease (NCD) worldwide is estimated to exceed USD47 trillion and create an increase in poverty levels [5], while a cumulative strain is expected to be placed on national economies and their healthcare systems. Chronic and degenerative conditions are a global epidemic, and mental health issues such as depression and anxiety are increasing. Both of these rose by 54% and 42%, respectively, from 1990 to 2013 [6]. In 2014 it was estimated that 700 million people worldwide had a categorized mental disorder of some kind [7].

In an Australian report by Cheema et al., non-communicable diseases (NCDs) were responsible for over 63% of the world’s annual death toll. More than 80% of these occurred in low- to middle-income countries during 2006–2012. CVDs, cancers, T2DM, and chronic kidney diseases are the leading NCDs globally. The foremost pathophysiological risk factors for these diseases include overweight and/or obesity, hypertension, and hyperglycemia. These impact economies extremely negatively [4].

Few real solutions to the global burden of NCDs have been proposed, but it has been recognized that incredible suffering could be avoided and trillions of dollars saved if a large proportion of the population were to engage in supportive health behaviors. If governments were to invest in strategies that promoted and rewarded health, this could come about quickly. By using the definition of health as a reference, “*a state of complete physical, mental and social wellbeing and not merely the absence of disease*” ([8], para. 1), the current global burden of disease is evidence that healthcare systems have failed to promote health. A shift of focus within healthcare from rehabilitation to prevention is required and urgently [9].

Five key areas were identified by the World Economic Forum report of September 2011 on the Global Economic Burden of NCDs ([5], p. 6)):

(1) NCDs are already an enormous economic burden and will change dramatically over the next two decades.
(2) Although high-income countries currently have the greatest economic burden of NCDs, the developing world, especially middle-income countries, is expected to take an ever-bigger portion as their economies and populations grow.
(3) CVD and mental health problems are the main contributors to the global economic burden of NCDs.
(4) The World Economic Forum’s annual Executive Opinion Survey (EOS), which is included in its Global Competitiveness Report, shows that nearly half of all business leaders surveyed worry that at least one NCD will hurt their company’s bottom line with similarly high levels of concern in the low-, middle- and high-income nations.
(5) There appear to be many options available to prevent and control NCDs–cost-effective models of care–models that reduce the burden of caring for the family by other family members who are not trained. More research into the benefits of these interventions versus their costs is required [5].

There is a significant body of evidence that supports the use of CEPs in the effective management of chronic conditions, one option already available to prevent and control NCDs which is cost-effective. In a lifestyle intervention study by Forsyth et al., conducted in NSW AUS for patients with mental illness, it was reported that cardiovascular fitness, muscle endurance, and psychological wellbeing improved in 80% of people who completed the program. More than 95% of respondents involved in a CEP coordinated healthy lifestyle program in Western AUS reported improved fitness, physical wellbeing, and mood [10], reducing the need for more expensive treatment options.

## 3. New Zealand Burden of Disease

According to the WHO NCD country profiles of 2018, 89% of NZ and AUS deaths are attributed to NCDs [11]. Table 1 indicates NZ vs. AUS mortality rates.

### 3.1. Cardiovascular Disease

CVD contributes to the highest rate of NCD mortality in both AUS and NZ. In NZ, this includes IHD at 16.3% (4600 people) and stroke at 7.6% (2100). It is the number one killer of women globally, and NZ women are five times more likely to die from CVD than breast cancer [12]. These numbers include mortality only and not the number of people living with long term CVD or those living with a disability after cardiac or cerebrovascular events [11]. The economic cost of this is substantial.

Stroke is the second primary reason for mortality in NZ after IHD, with around 2000 deaths per year [13]. Stroke is the primary cause of severe adult disability in NZ, and 9000 New Zealanders have a stroke every year. There are ±60,000 stroke survivors in NZ, and approximately 25% of these survivors are permanently disabled as a result. There is also a high incidence of depression, with up to 60% of patients having a period of subsequent depression post stroke [14]. The NZ Institute of Economic Research predicts the cost of stroke in NZ is approximately NZD1.1 billion for 2020. It is estimated to increase to NZD1.7 billion by 2038 [15]. NZ healthcare’s overall budget for 2020/21 is only $20.27 billion [16].

The number of people suffering from a stroke would be reduced by more than 50% if all suggested risk reduction strategies were accepted in the community. Typical risk factors for stroke include T2DM, smoking, a high salt intake, hypertension, and overweight [17]. Programs that consider behavioral and modifiable risk factors related to stroke at the primary health and community levels are the most effective way to reduce the increasing impact of stroke in NZ. Primary intervention programs would also, in addition, be likely to assist in managing and reducing other health problems, like CVD and T2DM. These interventions need to be taken into account and implemented as a priority [14]. CEPs can be integrated into the primary and community health multidisciplinary teams to implement supervised risk-specific reduction exercise prescription and intervention. Assessment of suitability for exercise interventions is paramount.

Table 2 indicates the leading causes of death within NZ, with IHD being the number one cause of death in three out of the four groups [18]. Maori are the indigenous Polynesian people of NZ. Note that T2DM only appears in one group. This may be because the complications of diabetes cause an increase in cardiac risk and these deaths could be reported as cardiac deaths.

The Annual Update of Key Results of 2018/2019 from the Ministry of Health, adjusting for age and population size, indicated that Maori health loss was nearly 1.8 times higher than non-Maori. More than half of this Maori health loss occurred before middle age. If Maori had experienced health loss at similar rates to non-Maori at all ages, the loss of health among Maori would have been 42% lower and health loss of the whole population 7% lower [18]. The need for intervention in these population groups to promote equity in health within NZ is imperative. Primary and community risk prevention programs would benefit from the addition of CEPs within these groups as a frontline means to address this inequity.

With NCD mortality almost identical to AUS, NZ needs to adopt the same or better intervention strategies to combat the unnecessary high death rate due to modifiable risk factors and the economic burden this places on the NZ healthcare system.

A 2017 review investigated the effects of exercise-based interventions on CVD risk factors in individuals with stroke or transient ischemic attack. Findings suggest that exercise-based interventions effectively reduce systolic blood pressure, fasting glucose and insulin, and improve high-density lipoprotein cholesterol, which provides evidence for their implementation as a strategy for secondary prevention [19]. An older but larger review, Heran et al. found that in coronary heart disease, exercise-based cardiac rehabilitation decreased cardiac mortality [20]. There was evidence of a notably higher level of quality of life with exercise-based cardiac rehabilitation than usual care in 7 out of 10 trials reporting health-related quality of life, which used validated measures [21].

Swedish researchers examined data from 22,227 heart attack patients between 2004–2013 over a six to ten-week period after their event. Those who stated they had increased their activity between two follow-ups had a 59% lower risk of dying over the next four years. The group that was constantly active had a 71% lower risk of death [22]. Sofi et al., (2008) conducted a study that confirmed noteworthy protection from moderate-to-high levels of physical activity against coronary heart disease [23]. Haykowsky et al., demonstrated the positive effect of exercise interventions on cardiac remodeling in congestive heart failure patients. In 14 trials that informed ejection fraction data in 812 patients, aerobic training considerably increased their ejection fraction [24]. “*Aerobic training is an inexpensive and effective non-drug, non-device, non-surgical intervention that helps to reverse or slow the progression of ventricular 5emodeling and improves VO2 peak in clinically stable individuals with systolic dysfunction*” ([24], p. 2335). Davies et al. showed with exercise therapy, heart failure-related hospitalizations were reduced, and health-related quality of life improved [25]. A systematic review by Roine et al. revealed that exercise intervention compared to usual care was found to be cost-effective for coronary artery disease [26].

CEPs are integral in both inpatient and outpatient cardiac rehabilitation scenarios. A CEP has the ability to determine the most effective testing protocol for a cardiac patient. It requires the CEP to have advanced knowledge in the absolute and relative contraindications to testing prescription, to determine the effectiveness of this protocol with each patient and adjust it where necessary. These decisions are made based on the ability and pre-existing conditions of each client.

In most cases, a patient with CVD will also have several other risk factors or conditions. CEPs are clinical experts who determine the effectiveness of exercise testing and programming, particularly regarding complex cases such as a combination of both CHF and T2DM. Each condition independently requires specific exercise intervention techniques. The CEP can make judgments based on which exercise protocols may be the most effective to both conditions and not detrimental to one in favor of progress in the other. In many instances, overweight and obesity with a secondary cardiac condition often include musculoskeletal conditions such as lower back pain and/or osteoarthritis of the knees and hips where patients may have had joint replacement surgery or be on a waiting list for one. CEPs are trained in both chronic disease and musculoskeletal rehabilitation, thereby eliminating the need for two separate practitioners to be involved in the entire rehabilitation process.

Exercise in the rehabilitation of CVD has an incremental cost per QALY saved of AUD42,535 [27], exercise is more cost-effective than stent angioplasty [28], and exercise interventions were more cost-effective than usual care for heart failure patients [29]. In the 2015 Deloitte Access Economics report, it was estimated that a consumer with CVD would obtain AUD12.10 in wellbeing benefits, better productivity, and reduced health system expenditure for every AUD1 spent on clinical exercise physiology exercise interventions [21]. A study of this magnitude needs to be made a priority in NZ.

### 3.2. Mental Health

Mental health disorders are the foremost contributor to disability among people of working age in developed countries [30] and one of the major health loss sources in NZ [11]. Mental illness/unwellness is also bi-directionally associated with additional comorbidities. Just as depression and anxiety are related to the increased risk of chronic disease, numerous chronic diseases increase the risk of depression and anxiety [31]. Anxiety, depression, and substance abuse are the most frequently diagnosed conditions.

The total NZ population has a 12-month prevalence of mental disorder burden of 20.7%, whereas pacific peoples (people from the Pacific Islands that now reside in NZ) sit much higher, at 25%. One-third of all individuals receiving health or disability benefits from the NZ government suffer from a mental health condition as their primary condition that affects their ability to work [32]. The 2019/20 NZ Health Survey reported Maori and European NZ’s mental health issues increased since 2011/2012. Maori adults were 1.9 times more likely to suffer mental distress as non- Maori adjusting for gender and age [33].

People with severe mental illness have more significant medical needs and hospitalization frequencies and are at higher risk of developing long-term CVD problems. They are also much more susceptible to premature death than the general population, nearly two to three times higher [34]. Two-thirds of this premature mortality is due to cancer, CVD, and additional physical conditions [35]. Advancing well-timed access to mental health services and interventions and averting mental health problems and illness is significant to improving the overall health of New Zealanders, given the strong correlation between mental illness, physical health, and premature mortality [35].

The National Institute for Health and Clinical Exercise guidelines for treating and managing depression in adults recommend that physical activity programs be implemented for people with persistent depressive symptoms or mild to moderate depression. They convey that these interventions should be delivered in groups with support from competent practitioners such as exercise physiologists. They should typically consist of three sessions of moderate duration over 10–14 weeks [36]. A systematic review by Nystrom, et al., recommended that exercise interventions be individually customized physical activity, performed under supervision when treating major depressive disorder [37]. In a systematic review and meta-analysis by Vancampfort, et al. on cardiorespiratory fitness among people with severe mental illness (SMI), exercise should focus on improving fitness to reduce all-cause mortality [38]. A recommendation from the review was that qualified professionals supporting people with SMI must be included as part of the multidisciplinary team. These qualified professionals are CEPs.

One in three Australian nurses working in mental health testifies that they consult with CEPs about their patients with mental illness and their state of physical health [39]. About 80% of general practitioners in the UK refer patients with depression for exercise intervention programs for treatment [39]. The Mental Health Intensive Care Unit (MHICU) at Prince of Wales Hospital in Sydney has incorporated exercise as a routine component of care [21].

Already in 2003, the Australian Institute of Health and Welfare determined that every case of depression prevented translated to 0.127 QALYs averted annually. This was extremely cost effective [40]. According to Deloitte Access Economics, for every AUD1, the CEP exercise intervention costs the patient; they would receive AUD10.80 in better wellbeing, productivity loss reductions, and health system costs. For patients with depression, this is highly valuable [21].

### 3.3. Diabetes

In 2017 it was estimated that 451 million adults were living with diabetes worldwide [41]. It is expected to rise to 693 million by 2045. The global healthcare expenditure on people with diabetes was projected to be USD850 billion. It was also estimated that 49.7% of people living with diabetes are undiagnosed, and an estimated 374 million people live with impaired glucose tolerance (IGT) [41]. It was estimated that some form of hyperglycemia during pregnancy affected almost 21.3 million live births. Virtually 5 million deaths globally were due to diabetes in adults, and the estimates of diabetes prevalence, deaths attributable to diabetes, and healthcare spending due to diabetes present significant financial, social, and health system burdens around the world [41]. In NZ, at the end of December 2019, 263,938 individuals were enrolled with a primary health organization (PHO) with either type 1 or 2 diabetes [33]. It is estimated that there are around 100,000 people who have undiagnosed diabetes [33]. The occurrence of diabetes is higher amongst those living in lower socio-economic areas, Maori, Pacific, and Indo-Asian populations [42].

Studies show that physical activity is beneficial for people with T2DM, but adherence to an exercise program and/or initiating physical activity can be challenging. Research published in the Journal of Physical Activity & Health conducted a systematic review evaluating the effects of structured exercise, behavioral interventions, and the correlation to long-term physical activity adoption in people with T2DM [43]. The review included 23 randomized control trials, which consisted of 9,640 participants whose exercise habits were tracked for at least six months. The findings showed that all five structured exercise trials improved physical activity compared to non-exercise controls. Of the behavioral interventions, a little over half improved physical activity, with greater effects seen when there was more face-to-face counseling or supervised exercise [43]. These results indicate that structured, supervised exercise may lead to greater physical activity adoption for periods longer than six months. The role of the CEP here is twofold. Firstly, to initiate behavior changes, improve adherence to physical activity by using structured, face-to-face, exercise therapy sessions, and address each patient’s needs.

The Deloitte Access Economics report agreed that there was enough clear evidence that demonstrated Clinical Exercise Physiology lifestyle interventions effectively managed and prevented type 2 diabetes. Enhanced insulin sensitivity, decreased weight, better glucose management, reduced blood pressure, total cholesterol, and triglycerides, as well as reduced heart disease risk, were the benefits of CEP intervention programs [21].

A UK review of exercise referral schemes in primary care by Campbell et al., stated that support and expertise of CEPs was a feature of the highly valuable interventions, as it assisted participants to become familiar with their own exertion levels as well as helping them to use exercise equipment safely and effectively. The CEP role in monitoring the improvement and endorsing progression was also valuable [44]. Therefore, studies show that specialized exercise therapists such as CEPs, offering science-based, supervised activities, are beneficial in two aspects, firstly pre-diabetes/T2DM prevention and secondly management of these conditions. The Deloitte report estimated that for every AUD1 a patient with T2DM spends on CEP interventions, they would receive AUD8.50 in reduced health care costs, improved wellbeing, and productivity loss reductions [21].

## 4. Economic Burden of Noncommunicable Disease in NZ

A report by the Ministry of Health from as far back as 2009 indicated, “*long term conditions are now the major challenge for the NZ Health system. Two in every three NZ adults have been diagnosed with at least one long term condition, and long- term conditions are the leading driver of health inequalities. Most of the studies estimated annual societal costs of more than NZD100 million per condition or risk factor*” ([45], p. 7). However, there has been very little risk reduction intervention for long term conditions over the last 11 years. Several studies directed over decades have reliably revealed that scientific exercise prescription involving effective approaches of aerobic and resistance training can avert and reverse T2DM, CVD, and overweight/obesity. These exercise interventions can also reduce risk factors, including hypertension, endothelial dysfunction, dyslipidemia, insulin resistance, depression, and systemic inflammation, contributing to these NCDs [4].

Nine of the top ten causes of mortality in high-income countries were due to long-term conditions. Long-term conditions also account for the top nine causes of disease burden (DALYs) in high-income countries [11].

Three approaches can be utilized to calculate health problems’ economic burden ([5], p. 14):

(1) The cost of illness approach (COI) sums up both the overall direct and indirect costs of a disease, such as medications, diagnostics, procedures, inpatient and outpatient care, transport, information, research, pain and suffering, and sometimes income losses.
(2) The value of lost output estimates the effect of the disease on the GDP directly linked to loss of labor, production, and capital.
(3) The value of a statistical life year approach (VSLY) places an actual value on the year of life lived by a person and incorporated the willingness to pay to reduce disability, and goes beyond the GDP approach

The VSLY approach includes the use of DALYs or disability-adjusted life years, defined as one lost year of a healthy life. “*The sum of these DALYs across the population, or the burden of the disease, can be thought of as a measurement of the gap between current health status and an ideal health situation where the entire population lives to an advanced age, free of disease and disability*” ([46], p. 1). Therefore, a Quality Adjusted Life Year (QALY) is defined as one year of ideal health [11].

Diabetes NZ and Pricewaterhouse Coopers (2001, 2007, 2008) completed three studies that estimated the cost of publicly provided health services for T2DM. The estimated direct costs for T2DM in 2001 of NZD247 million, NZD540 million in 2007, and NZD600 million in 2008 [47]. The PricewaterhouseCoopers 2007 study also estimated direct costs of obesity in 2004 at NZD460 million and indirect costs due to lost productivity of NZD370 million [45].

The WHO estimates that total Healthcare costs in NZ in 2014 was NZD4018 per person with a gross national income average of NZD30,750. This is 11% of the GDP. In 2012 it was only NZD3721 pp per year with a gross national income average of NZD31,542. This is an increase of 8% over two years, with an inflation rate estimated at only around 1.9% for the third quarter of 2018 [48].

In its Social Cost of Road Crashes and Injuries Report 2019 update, the NZ Department of transport estimated the value of a statistical life year (VSLY) or VOSL as per their report NZD 4.56 million per life lost [49]. The social cost is estimated based on the following criteria:

- Death or loss of quality of life
- loss of output due to temporary incapacitation
- medical costs
- legal costs
- costs of vehicle damage costs

Due to NCD costs, chronic disease and disability are also based on the same first three listed criteria except that the loss of output due to temporary incapacitation becomes a loss of output based on permanent incapacitation with chronic cardiovascular, metabolic and mental health issues. The inability of a person to work and contribute to the country’s economic growth while still burdening the country with medical, disability, and other expenses is many of the costs associated with DALYs.

Robinson et al., in 2017, estimated the VSLY at three times the national GDP per capita [50]. In 2018 the world bank estimated NZ’s GDP at USD41,945 per person. This equates to USD125,835 per VSLY. If the 2026 estimated DALYs in NZ sits at 1.23 million, this equates to over USD154 billion in revenue lost due to disease and disability. According to the NZ. Ministry of Health, 70–80% of this burden is avoidable through a combination of prevention and treatment [45].

## 5. Discussion

The evidence suggests that CEP supervised exercise programs for managing CVD, mental illness, and T2DM are incredibly cost-effective and can result in large reductions in health system costs. Although there is substantial evidence to support the positive health effects and increase in QALYs of CEP intervention programs in many chronic disease conditions worldwide, there is little evidence on the economic benefit of CEP intervention programs within NZ. The fact that AUS and NZ share similar NCD profiles and healthcare systems could imply that NZ would benefit economically from CEPs being recognized health care practitioners. Developing CEP-led exercise and NCD intervention strategies that address the reduction of NCD’s economic burden and address the need for improved health outcomes in Maori and Pacific peoples, particularly, is imperative within NZ. This is a future area of research that needs to be led by CEPs in NZ.

Multidisciplinary interventions must endorse engagement and retention of obese patients [51] as NZ now has the third-highest occurrence of overweight and obesity in the OECD. It is acknowledged that obesity is the leading risk factor creating health loss in NZ [42] and is directly related to CVD and T2DM. The 2014 Canterbury District Health Board findings from interventions for obesity conducted in NZ confirm international literature that states individualizing and regular continued contact enhance adherence and effectiveness of interventions [52]. Program content, setting, and delivery must be individualized. Maori, Pacific, and other health models need to be merged to support and guide wider health and cultural beliefs and provide the opportunity for including family and community support. These models need to be tailored to the individual so that the intervention effectiveness is enhanced, specific diet and physical activity behaviors are directed, social support is available, and firm behavior change practices are used. They need to be pro-equity, affordable, and easily accessible for both patients and referring practitioners [52]. “A practical strategy may be for health professionals to work alongside their patients to choose an evidence-based intervention that aligns with their personal values and can be tailored to their lifestyle and abilities” [52], (p. iv–v). These areas all fall within the clinical expertise of the CEP.

“Living well, staying well and getting well” are the three key concepts the NZ Ministry of Health has made its primary focus in its ongoing vision for a healthier NZ [53]. He Korowai Oranga- the Maori Health Strategy, supports the Ministry of Health and DHB to improve Maori health oucomes [53]. There is no evidence that CEPs have been included within this funding model despite the large improvement in health and the economic impact they can make within this demographic.

‘Ala Mo’ui- Pathways to Pacific Health and Well-being is a strategy to improve Pacific peoples’ health and wellbeing, address life expectancy, health expectancy, and reduce ambulatory sensitive hospital (ASH) admission rates in the Pacific population within NZ. The original plan spanned four years from 2010–2014 to supply high-quality health services that would meet Pacific people’s needs. It was successful, and a second term from 2014–2018 followed [54]. Although the key health system and health status indicators are long-term measures, it is imperative that all three indicators reflect positive health trends for Pacific families and communities in order to reach equity in health outcomes [54]. CEPs would be best placed to influence life expectancy, health expectancy, and reduce ASH rates.

The Primary Healthcare Strategy was developed in 2001 and included the establishment of PHO’s, which set the direction for NZ’s health care. The NZ. Health Strategy of 2016 highlights the need for a move away from treatment to prevention and improve people’s lives. The main aim is to support improved financial sustainability [53]; CEPs that are health professionals that improve mortality outcomes and quality of life utilizing scientific exercise prescription while reducing economic burden.

Rising to the Challenge looks to enhance outcomes for patients, their families, and the supportive community who utilize primary and/or specialist mental health and addiction services. It offers planners, funders, and providers who deliver government-subsidized mental health services with some direction in terms of priority areas for service development [55]. Although there has been some implementation of physical activity provided for this program, and there is clear evidence to motivate for structured and individualized intervention, the preferred providers should be CEPs.

Living Well with Diabetes is a strategy for individuals with a high risk of developing T2DM or those living with the disease. The health sector has attempted to recognize those at risk of acquiring diabetes sooner and advance the quality of services for people who already suffer. The government states it is dedicated to maintaining a systematic approach that is sustainable in order to reduce the burden of diabetes and the associated comorbidities in NZ [56]. CEPs must be included in this healthcare model as there is evidence that individualized lifestyle interventions early on are effective in both the management and prevention of T2DM.

Green Prescription (GRx) is when general practitioners prescribe physical activity formally to patients as a cure or for management of an existing medical condition, or as a tool for preventing disease/disability. Patients can access advice and support as well as subsidized physical activities. Sixteen providers deliver GRx to patients and/or families that have been referred to. There are two primary health organizations (PHOs) and fourteen regional sports trusts [57]. Some of these organizations employ CEPs to manage their high-risk patient profiles. CEPs would be best placed to run the Green Prescription programs, funded by the Ministry of Health, within their practices based on the evidence supporting their success in disease management and prevention in AUS and the UK.

CEPs need to be utilized to improve health outcomes and reduce NCDs’ economic burden within NZ.

There is also a dire need for Maori and Pacific CEPs to support the underlying themes of both the He Korowai Oranga and the ‘Ala Mo’ui. The advancement of more Pacific undergraduate health and sports science students towards postgraduate CEP qualification is necessary to align with the four priority outcomes of ‘Ala Mo’ui. The same is true of the need for more postgraduate Maori trained specialists within the field of clinical exercise physiology, which would directly influence the Maori health model of Te Whare Tapa Wha.

In a paper to evaluate the application of kaupapa (principle/policy) in lab-based research by Warbrick et al., the discussion of the application of, and an interface between, kaupapa Maori methods of research and those traditionally used in exercise physiology was explored. The paper concluded that exercise science has the potential to bridge indigenous and western approaches to research, informing both the prevention and the treatment of lifestyle illnesses that impact significantly on Maori communities [58].

## 6. Conclusions

A CEP holds a postgraduate tertiary qualification. They are allied health professionals specializing in delivering scientific exercise interventions for acute, subacute, or chronic medical conditions. These conditions include but are not limited to cardiovascular, respiratory, endocrine, musculoskeletal, mental, and neurological diseases.

According to Australian research, CEPs’ interventions have extensive benefits for chronic conditions such as CVD, Mental illness, and T2DM. The benefits include reduced health system expenditure, improved productivity, and improved wellbeing, and this is by far the greatest benefit patients will encounter. CEPs are an immensely underutilized tool in the multidisciplinary care and treatment of those with chronic disease [21]. The CEP can also make judgments based on which exercise protocols may be the most effective to a variety of comorbidities and conditions and not detrimental to one in favor of progress in the other.

There is more than enough evidence to support the fact that CEP interventions improve physical outcomes in a variety of chronic disease patients, and the cost to benefit ratio has been clearly shown in AUS. The overall lifetime burden of disease savings in exercise interventions in people with CVD, Mental illness and T2DM by CEPs is substantially higher than the cost of the intervention programs they offer.

As both AUS and NZ use similar healthcare models, there is a possibility that CEPs could be merged into the NZ. National Health Strategy, He Korowai Oranga, and ‘Ala Mo’ui in the same format as Medicare. Involvement in programs such as Green Prescription, Living Well with Diabetes and Rising to the challenge, would also assist in reducing the burden of these conditions on the NZ health care system, opening resources for better prevention strategies in the future. Further investigation into the cost-effectiveness of CEP intervention strategies is therefore required within a NZ context.

This review aimed to identify the areas that CEPs can make a difference both to the survival and long-term illness outcomes of the NZ community, and further investigation and study in this area is imperative. This cannot be done without funding. The following three areas frame the bigger picture:

(1) Prevention is better than a cure
(2) Effective, evidence-based treatment should be used at all costs during disease management and treatment
(3) Cost-effectiveness is key

The NZ. Ministry of Health needs to address wellness more proactively by allocating funding to evidence-based practice that reduces NCD economic burden in NZ. The extensive evidence currently available to the NZ government, private healthcare insurers, and primary healthcare should inform strategy to overcome the barriers to utilizing clinical exercise physiology services.

## Figures and Tables

**Table 1:** NZ vs. AUS NCD’s 2018. Adapted from the WHO NCD Country profiles of 2018 [11].

	New Zealand	Australia
Total NCD Mortality	89%	89%
Cardiovascular Disease	31%	28%
Cancer	30%	29%
Chronic Respiratory Disease	7%	7%
Diabetes	3%	3%
Other NCDs	19%	23%

**Table 2:** Leading causes of Death for Maori and Non-Maori [18].

	Male	Female
**Maori**	Ischaemic heart disease	Lung cancer
	Lung cancer	Ischaemic heart disease
	Suicide	Chronic obstructive pulmonary disease
	Diabetes	Cerebrovascular disease (stroke)
	Motor vehicle accidents	Diabetes
**Non-Maori**	Ischaemic heart disease	Ischaemic heart disease
	Suicide	Breast cancer
	Lung cancer	Cerebrovascular disease (stroke)
	Cerebrovascular disease (stroke)	Lung cancer
	Motor vehicle accidents	Colorectal cancer

## Data Availability

Not applicable.
